# Antioxidant supplements and semen parameters: An evidence based review

**Published:** 2016-12

**Authors:** Sedigheh Ahmadi, Reihane Bashiri, Akram Ghadiri-Anari, Azadeh Nadjarzadeh

**Affiliations:** 1 *Nutrition and Food Security Research Center, Shahid Sadoughi University of Medical Sciences, Yazd, Iran.*; 2 *Diabetes Research Center, Shahid Sadoughi University of Medical Sciences, Yazd, Iran.*

**Keywords:** *Male infertility*, *Semen*, *Antioxidants*, *Carnitine*, *Coenzyme Q10 (CoQ10)*, *Vitamins*

## Abstract

Many studies have focused on male infertility. There is limited evidence about the influence of nutrition on quality of semen. Approximately, 30-80% of infertility cases are caused by oxidative stress and decreased level of seminal total antioxidant capacity. This study was aimed to review the effects of oral antioxidant supplements on improving major semen parameters such as sperm concentration, motility, morphology, DNA damage, and fertility rate. Data were extracted from PubMed and Google scholar database by using the terms “antioxidant”, “multivitamin”, “carnitine”, “CoQ10”, “vitamin C”, “vitamin E”, “zinc”, “folic acid”, “N-acetyl cysteine” and “selenium” combined with “male infertility”, “semen”, and “sperm” to generate a set of relevant citations. Supplements such as CoQ10 and alpha-tocopherol significantly improve sperm count. Also, carnitine has positive effects on sperm motility and morphology. Simultaneous administration of vitamin E and vitamin C reduces the sperm DNA damage. However, in some studies, one or more factors have not changed substantially. In most of the studies, antioxidant supplementation improved the number, motility, morphology and sometimes DNA integrity of sperm. The present study showed that antioxidant supplements, especially a combination of antioxidants such as vitamin C, vitamin E, and CoQ10 intake can effectively improve semen parameters in infertile men.

## Introduction

Infertility is defined as not being able to get pregnant despite having frequent, unprotected sex for at least a year (1). More than 70 million couples suffer from infertility worldwide. Male infertility is a controversial issue throughout the world. Between 8 and 12% of couples suffer from infertility, based on research (2). Male factors account for at least 50% of all infertility cases worldwide (3). Some factors such as radiation, smoking, varicocele, infection, urinary tract infection, environmental factors, nutritional deficiencies and oxidative stress contribute to male infertility (4, 5). Oxidative stress occurs when the production of reactive oxygen species (ROS) exceeds the body’s natural antioxidant defenses (6). 

The increased level of ROS can be resulted from environmental factors such as high temperature, electromagnetic waves, air pollution, insecticides, alcohol consumption, obesity and poor nutrition (7). There are evidence that sperms are simply affected by ROS and oxidative stress. There are a number of studies that support the role of ROS in male infertility theory (8-11). The structure of the plasma membrane is unique and consists of high levels of polyunsaturated fatty acids (PUFAs) that improve membrane flexibility. It makes them vulnerable to be attacked by ROS (12-14). Lipid peroxidation cascade can seriously compromise the functional integrity of membrane cells, decrease sperm motility, and subsequently reduce fertility. ROS production pathologically results in high levels of DNA damage that is associated with properties of mitochondrial membrane (15). 

Ordinary antioxidants in semen include vitamin E, vitamin C, superoxide dismutase, glutathione and thioredoxin. These antioxidants neutralize free radical activity and protect sperm from ROS that already produced (6). Evidence show lower antioxidant capacity of semen in infertile men that explain the reduced semen antioxidants and high levels of ROS compared to fertile men (16, 17). Semen analysis may identify and characterize the following impairments in male: oligozoospermia (low concentration of sperm), asthenospermia (reduced sperm motility), teratozoospermia (sperms with abnormal morphology), and the combination them (oligoasthenoteratozoospermia) (18). Standard values of sperm parameters are as follow: pH equal or greater than 7.2, semen concentration equal or greater than 15 million per milliliter, and semen volume 1.5 ml or higher, sperm progressive motility of 32% or higher, and normal morphology equal or more than 4% (18). 

In recent years, most attention is on the effect of oxidative stress on the etiology of male infertility and the role of oral antioxidant supplements in improvement of semen properties in infertile men. Majority of these studies show a positive relationship between antioxidants and improved male infertility. However, some studies revealed paradoxical results. The aim of this study was evaluating the effect of antioxidant supplements on key semen parameters such as sperm concentration, motility, morphology DNA damage, and fertility rate. 

Data about effects of antioxidants on improved sperm quality were extracted from papers published between 2004 and 2015 in electronic databases from two sites, PubMed and Google scholar using the following keywords: antioxidant, multivitamin, carnitine, CoQ10, vitamin C, vitamin E, zinc, folic acid, N-acetyl cysteine, selenium and male infertility, semen and sperm. Searches were limited to the title and abstracts of clinical trials and meta-analysis. Animal and laboratory studies were excluded from the study. Also, studies that focused on the effect of antioxidants in combination with drugs were excluded to determine the positive effects of antioxidant supplements. Table I shows the clinical trials presented in this study.

**Vitamin C and vitamin E**

Ascorbic acid known as vitamin C is a water-soluble antioxidant that acts as a key cofactor in various hydroxylation and amidation processes (19). It is utilized in the synthesis of collagen, proteoglycan, and components of the intercellular matrix along with vitamin E (20). Vitamin C can be found in high concentrations in seminal plasma (21, 22). As vitamin C intake increases its concentration in seminal plasma rises and prohibits DNA damage (23). Vitamin E is a fat-soluble antioxidant that neutralizes free radicals and protects cellular membrane against O2 free radicals. It also prevents lipid peroxidation and therefore improves functions of other antioxidants (24). Vitamin E also inhibits the production of ROS in infertile male (25). 

Gerco *et al* conducted an interventional study on infertile men. Intervention group was treated by 1 gr of vitamin E and 1 gr of vitamin C. After two months, level of DNA damage was reduced in intervention group (p<0.001). However, no significant relationship was found between vitamin E and C intake and major semen parameters such as motility and concentration (26). Results from intra-cytoplasmic sperm injection (ICSI) and IVF show that high levels of sperm DNA damage results in lower fertility rate or infertility. Greco and Colleagues stated that two-month treatment with 1gr vitamin E and C improved ICSI success rate in patients with sperm DNA damage and reduced the level of DNA damage in these individuals (27). Moslemi *et al* studied 690 infertile men with idiopathic asthenoteratospermia who received daily supplement of selenium (200 μg) in combination with vitamin E (400 IU) for at least 100 days. They reported 52.6% (362 cases) total improvement in sperm motility, morphology, or both, and 10.8% (75 cases) spontaneous pregnancy in comparison with no treatment (28).

**Carnitine**

L-carnitine (LC) or 3-aminobutyric acid is a naturally occurring compound and also a semi-essential vitamin like substance required for human metabolism. LC involvement in intermediary metabolism is essential for bioenergetic processes, where it has a major role in the formation of acyl carnitine esters of long-chain fatty acids (29). The highest concentrations of LC exist in epididymis which is 2000 times higher than whole blood concentration (30, 31). The high level of LC in epididymis is resulted from an active secretory process (29). Findings show a positive relationship between initial sperm movement and increased LC in epididymis and L-acetyl in sperm (30, 32). 

Some studies have examined the effect of L-carnitine supplementation on male infertility. Lenzi *et al* conducted a double-blind controlled clinical trial to evaluate the effect of LC on male infertility. A total of 60 infertile men with oligoasthenoteratozoospermia were divided into two groups (intervention and control group). Intervention group received 2 gr/day LC and 1 gr/day L-acetyl carnitine (LAC) for 6 months of therapy. A positive relationship was observed between LC and LAC and sperm motility in infertile men. However, this relation was more significant in men with lower sperm motility at the baseline (33). 

Balercia *et al* evaluated the effect of LC and LAC or combined LC and LAC on the semen motion kinetics and total oxygen radical scavenging capacity (TOSC). This randomized double-blind controlled trial consisted of 60 men with idiopathic asthenoteratospermia. A six-month intervention showed that LC and LAC increased sperm motility and TOSC in men with asthenoteratospermia. Nine pregnancies occurred in carnitine-treated patients during therapy and that five of them were achieved after combined LC plus LAC administration (34). Sigman *et al* found no significant positive relationship between LC and LAC therapy and sperm motility and sperm concentrations, and no statistical difference was observed between two groups (35). 

Garolla *et al* examined the effect of LC therapy and Phospholipid hydroperoxide glutathione peroxidase (PHGPX) therapy in men with asthenoteratospermia. A total of 30 men with idiopathic asthenoteratospermia underwent this double-blind study and were divided into two groups based on PHGPX levels. Patients received a placebo for 3 months, and then received LC 2 gr/day daily for 3 months, too. Semen analysis showed that LC therapy improved sperm motility in patients with normal PHGPX levels (36). Wu indicated that short-term administration of LC can positively affect sperm count and leading to successful pregnancy through ICSI (37).

**Coenzyme Q10 (CoQ10)**

CoQ10 also known as ubiquinone is an antioxidant. As a component of the electron transport chain, it participates in aerobic cellular respiration, which generates energy. This oil-soluble, vitamin-like substance is present in cell membrane and lipoproteins (38). In recent years, the role of this vitamin-like antioxidant in male infertility has been discussed widely. Balercia and colleagues examined the effect of CoQ10 on sperm motility in infertile men, which 60 men with idiopathic asthenoteratospermia received CoQ10 therapy in a double-blind controlled trial. 

After 6 months therapy, CoQ10 increased in the semen of patients who received CoQ10, and the sperm motility was improved in these individuals. Twelve spontaneous pregnancies were occurred (39). Another double-blind controlled intervention by Safarinejad *et al* on 228 unexplained infertile men with abnormal sperm concentration, motility and morphology, showed that 28 wk treatment with ubiquinone led to improvement in sperm density, sperm motility and sperm morphology in the intervention group compared to the control group (40). Nadjarzadeh *et al* conducted a double-blind placebo controlled clinical trial on 47 infertile men with oligoasthenoteratozoospermia (OAT). 

They were randomly assigned to receive 200 mg CoQ10 daily or placebo during a 16 wk period. The trial showed non-significant changes in semen parameters such as density, motility or morphology in CoQ10 group, whereas total antioxidant capacity was increased significantly (p<0.05) (41). They showed that three-month supplementation with CoQ10 increased catalase and superoxide dismutase (SOD) in semen of OAT men compared with control group. There was a significant positive correlation between CoQ10 concentration and normal sperm morphology, and also catalase and SOD concentrations. A significant difference was shown in seminal plasma 8-isoprostane in two groups (p=0.003) after supplementation, too (42). 

Finally, it was found that the concentration of CoQ10 was correlated with key semen parameters such as sperm concentration, motility and morphology because the total antioxidant capacity improves. Thakur suggested that daily administration of 150 mg CoQ10 improved semen parameters in infertile men (43). A meta-analysis showed that supplementing infertile men with CoQ10 does not increase live birth or pregnancy rates, but there is a global improvement in sperm parameters such as sperm concentration and motility and CoQ10 concentration in semen (8).

**Zinc**

Zinc is the second most abundant metal in the body after iron. Although red meat, fish and milk are rich sources of zinc, the world health organization suggested that zinc deficiency affects about one-third of the world's population (44). It has been shown that zinc supplementation normally protects the spermatozoa against bacteria and also prevents damage to chromosomes (45, 46). Zinc plays an important role in testicular development and sperm maturation (47). 

Zinc deficiency is positively associated with male hypogonadism and incomplete development of sex characteristics in humans (48). Decreased levels of zinc in the semen were associated with reduced sperm fertilization capacity (49). Ebisco and colleagues revealed that patients who received 5 mg of folic acid and 66 mg of zinc for 26 wks reported improving sperm concentration. However, no improvement was observed in other semen parameters. Furthermore, at baseline, positive correlations were found between serum Zinc and sperm concentration, motility and Inhibin B (50).

Hadwan and colleagues examined the effect of zinc supplementation on quantitative and qualitative characteristics of semen and ligands attached to the zinc in men with asthenoteratospermia. A total of 37 fertile male and 37 infertile men that have been adjusted for age, received 2 zinc sulphate capsules (220 mg per capsule) per day for 3 months. Results showed that the volume of semen, progressive sperm motility percentage and total normal sperm count increased after zinc supplementation. Zinc in seminal plasma binds with 3 types of protein (low molecular weight ligands, average molecular weight ligands and high molecular weight ligands). In this study, the percentage of high molecular weight ligands in the semen was higher in fertile men than in infertile men, and zinc supplementation increased the percentage of high molecular weight ligands in men with asthenoteratospermia and raised low molecular weight ligands above the normal value (45). 

Raigani *et al* did not show significant improvements in sperm concentration, motility and morphology after supplementation with folic acid, zinc, and combination of them for 16 weeks (51). Hadwan *et al* also examined the effect of zinc supplementation on the peroxynitrite levels, arginase activity and nitric oxide (NO) synthase activity in seminal plasma in men with asthenospermia. They concluded that peroxynitrite levels and NO synthase activity were significantly higher in the infertile patients compared to the fertile group. Peroxynitrite levels, arginase activity and NO synthase activity of the infertile patients were restored to normal values after treatment with zinc sulfate (46).

**Selenium and N-acetyl-cysteine**

Selenium is an essential trace element in formation of sperm and testosterone biosynthesis (52). At least 25 selenoproteins have been identified in humans and animals. Selenoproteins help maintain normal sperm structure integrity. N-acetyl cysteine is a naturally occurring compound which comes from amino acid L-cysteine, and functionsas a precursor of glutathione peroxidase (53). Placebo controlled clinical trial carried out in Iran and Tunisia showed that selenium supplementation improved sperm counts, concentration, motility and morphology as well as sperm concentration in infertile men (54, 55). 

Safarinejad *et al* investigated the effect of selenium and N-acetyl-cysteine on 468 infertile men with idiopathic oligo-asthenoteratospermia. They were followed by a 30 weeks treatment period. In response to treatment, serum follicle-stimulating hormone decreased but serum testosterone and Inhibin B increased. In addition, all semen parameters significantly improved with selenium and N-acetyl-cysteine treatment. Administering selenium plus N-acetyl-cysteine resulted in further beneficial effects in semen parameters (55).

**Multi** **-** **antioxidant supplementation**

Currently, multi-antioxidant supplementa-tions are considered as an effective therapy for male infertility. The synergetic effect of multi antioxidants made them interesting for researchers. Galatioto *et al* conducted a study to determine the effectiveness of an antioxidant therapy in the quality of seminal fluid parameters and the natural pregnancies in spouses of men with persistent oligospermia (5-20 million/ml) 6 months after retrograde embolization. 

20 men with varicocele received antioxidant therapy; NAC and vitamins-minerals (containing vitamin C, vitamin E, vitamin A, thiamine, riboflavin, biotin, B12, magnesium, ferrous, manganese, copper, zinc). After this therapy, significant statistically increases were found in number of sperms due to WHO index in treated groups. Also, no significant relationship was observed between multi-antioxidant supplementation and other semen parameters like motility and morphology. No spontaneous pregnancy was occurred after 12 months (56). 

Abad and colleagues also carried out a study to determine the effect of oral antioxidant treatment upon the dynamics of sperm DNA fragmentation following in a cohort of 20 infertile patients diagnosed with asthenoteratozoospermia. All subjects received 1500 mg of LC, 60 mg vitamin C, 20 mg CoQ10, 10 mg vitamin E, 10 mg zinc, 200 microgram folic acid, 50 microgram selenium, and 1 microgram vitamin B12 during a period of 3 months. Results showed that a proportion of DNA degraded sperm was significantly reduced and semen analysis data showed a significant increase in concentration, motility, vitality and morphology parameters. Also a significant improvement of DNA integrity at all incubation points was observed. Findings of this study suggest that antioxidant treatment improves sperm quality not only in terms of key seminal parameters and basal DNA damage, but also helps to maintain DNA integrity. Therefore, administration of antioxidants can help in new medical treatments (57). 

Gopinath stated that administration of antioxidants lead to a significant improvement in sperm count and sperm total motility at 90 days in men with oligoasthenoteratozoo-spermia compared with placebo (58). Tremellen *et al* conducted a prospective randomized double-blind placebo-controlled trial in sixty couples with severe male factor infertility. Participants were randomly assigned to take either one capsule per day containing 6 mg Lycopene, 400 IU vitamin E, mg vitamin C, 25 mg zinc, 26 microgram selenium, 5 mg folate and 1000 mg garlic or taking a placebo for three months prior to their partner's IVF or ICSI cycle. The antioxidant group recorded a statistically significant improvement in viable pregnancy rates (38.5%) compared to the control group (16%). No significant changes in oocyte fertilization rate or embryo quality were detected between the antioxidant and the placebo groups (59).

**Table I:** Characteristics of clinical trials reviewed in the study

**Author** **Year**	**Number of participants/ Abnormality**	**Antioxidant type and dose**	**Intervention period**	**Controlled/ Blinded**	**Results**
Lenzi 2004 (33)	60/ Oligoasthenoteratozoospermia	2 g/d LC plus 1 g/d LAC	6 months	Yes/ Yes	Increase in sperm motility
Balercia 2005 (34)	60/ Asthenozoospermia	a) 3 g/d LC b) 3 g/d of LAC c) 2 g/d of LC and 1 g/d LAC d) Placebo	6 months treatment and 1 month follow up	Yes/ Yes	LCand LAC increased sperm motility and TOSC. Nine pregnancies occurred in carnitine-treated patients during therapy and five of them were achieved after combined supplement
Greco 2005 (26, 27)	64/ Unexplained infertility with high DNA fragmentation	a) 1g/d vitamin E and 1g/d vitamin C b) Placebo	2 months	Yes/ Yes	No significant relationship was found between vitamin E and C intake and sperm motility or concentration but improved ICSI in patients with sperm DNA damage and reduced the level of DNA damage.
Ebisch 2006 (50)	47/ Fertile 40/ Subfertile	a) 5 mg folic acid and 66 mg zinc b) Placebo	26 weeks	Yes/ Yes	Improvement of sperm concentration with no effect on other parameters
Sigman 2006 (35)	21/ Asthenozoospermia	2 g/d LCplus 1 g/d LAC	24 weeks	Yes/Yes	No significant effect of LC / LAC and sperm motility / concentrations
Galatioto 2008 (56)	42/ Oligospermia	a) 600 mg NAC plus vitamins-minerals b) no treatment	3 months	Yes/ No	Increase in number of sperms in intervention group with no differences in other semen parameters
Balercia 2009 (39)	60/ Asthenozoospermia	a) 200 mg Co Q10 b) Placebo	6 months	Yes/ Yes	Improvement in sperm motility and twelve spontaneous pregnancies
Safarinejad 2009 (55)	468/ Oligoasthenoteratozoospermia	a) 200 µg selenium, b) 600 mg N-acetyl-cysteine, c) 200 µg selenium+ 600 mg N-acetyl-cysteined) Placebo	30 weeks	Yes/ Yes	All semen parameters significantly improved with selenium and N-acetyl-cysteine
Nadjarzadeh 2014 (41)	47/ Oligoasthenoteratozoospermia	a) 200 mg Co Q10 b) Placebo	3 months	Yes/ Yes	Increase in seminal catalase and SOD with a significant positive correlation between CoQ10 concentration and normal sperm morphology, catalase, and SOD
Safarinejad 2012 (40)	228/ Oligoasthenoteratozoospermia	a) 200 mg Ubiquinone b) Placebo	26 weeks	Yes/ Yes	Improvement in sperm density, motility and morphology
Moslemi 2011	690/ Asthenoteratospermia	200 μg Selenium + 400 IU vitamin E	100 days	No/ No	52.6% total improvement in sperm motility, morphology, or both with 10.8% spontaneous pregnancy
Hadwan 2012 (45)	37/ Fertile 37/ Asthenozoospermia	220 mg zinc sulfate bid	3 months	Yes/ Yes	Increase in semen volume, sperm count and motility
Raigani 2014 (51)	83/ Oligoasthenoteratozoospermia	a) 220 mg folic acid and 5 mg zinc b) placebo	16 weeks	Yes/ Yes	No significant improvements in sperm concentration, motility and morphology
Abad 2013 (57)	20/ Asthenoteratozoospermia	1500 mg of LC, 60 mg vitamin C, 20 mg Co Q10, 10 mg vitamin E, 10 mg zinc, 200 µg folic acid, 50 µg selenium, and 1 µg vitamin B12	3 months	No/ No	Significant increase in concentration, motility, vitality and morphology of sperm. Also a significant improvement of DNA integrity was observed.
Nadjarzadeh 2011 (42)	47/ Oligoasthenoteratozoospermia	a) 200 mg Co Q10 b) Placebo	3 months	Yes/ Yes	Improvement in seminal oxidative defense but does not affect on semen parameters in idiopathic oligoasthenoteratozoospermia
Hadwan 2014 (46)	60/ Fertile 60/ Asthenozoospermia	220 mg zinc sulfate bid	3 months		**Increase in semen volume, forward motility and sperm count**

LC: L-carnitine

LAC: L-acetyl carnitine,

TOSC: total oxygen radical scavenging capacity

SOD: Superoxide dismutase

## Discussion

It is believed that oxidative stress significantly affects male infertility. The present study reviewed randomized studies from 2004 to 2015. All studies that focused on the effect of LC on sperm key parameters showed a positive relationship between LC and semen parameters such as sperm count, motility and morphology (30, 32, 35). One study showed no significant relationship between LC therapy and semen parameters in respect to the smaller sample size compared to other studies (34). A meta-analysis by Lafuente showed that treatment with coenzyme Q10 led to a significant improvement in the sperm motility and density, whereas no significant improvements was observed in live birth and pregnancy rates (8). The combination of vitamin E and vitamin C showed no improvement in either sperm count or motility, but reduced sperm DNA damage (25). 

A number of studies have shown that combined selenium and N-acetyl-cysteine therapy improved male infertility (52-54). Moslemi found that combination therapy with selenium and vitamin E significantly improved sperm motility and morphology. Although, the sample size was large, this study lacked the control group (28). Two studies showed no improvement in sperm motility and morphology after zinc therapy (46, 49). However, two other studies showed a significant improvement in zinc-attached ligands and semen key enzymes after zinc therapy (44, 50). 

All studies which examine the effect of multiple antioxidants in a supplementation showed an improvement in semen parameters after therapy (56, 58). For example, combination therapy with carnitine, CoQ10, vitamin E and vitamin C for three to six months improved sperm concentration (60). Only one study showed a significant improvement in sperm concentration after combination therapy without improvement in motility and morphology (55). 

## Conclusion

This study reviewed a number of meta-analysis studies. We concluded that although majority of reviewed studies showed significant association between antioxidant supplementations and one or two semen parameters but administration of supplementations like L-carnitine, selenium, vitamin C and vitamin E may lead to improving sperm concentration, motility and morphology, and sometimes DNA integrity. Finally, this study suggest that further research should be done to determine the appropriate antioxidant compounds as well as certain dose of antioxidants in clinical practices. Moreover the future studies should concern the pregnancy rate as a primary outcome in their designs.

## Conflict of interest

There is no conflict of interests in this study.
